# A Case of Habitual Regurgitation, Simulating Rumination in Lower Animals

**Published:** 1874-03

**Authors:** D. W. Graham


### A CASE OF HABITUAL REGURGITATION, SIMULATING RUMINATION IN LOWER ANIMALS.

REPORTED TO THE CHICAGO MEDICAL SOCIETY, MEETING OF JANUARY 19TH, 1874, BY D. W. GRAHAM, M.D.

MR. D., aged twenty-four; an intelligent mechanic; of robust appearance; medium height; sanguine temperament; with muscular system exceedingly well developed; has been strictly temperate in all his habits; appetite and digestion have always been good, but never relished fatty articles of food; intestinal functions perfectly normal; has always had good health; family history good, except some manifestations of tuberculosis, on the maternal side. A suspicion of the development of this affection in himself, induced by a transient attack of bronchitis, led him to apply for an examination. A thorough physical examination gave no evidence of any organic disease of the lungs or heart.

In this connection, he mentioned that he was in the habit of regurgitating and remasticating his food, and wished to know the cause of it, and if there was any way of being relieved of the difficulty. He introduced the subject with apparent reluctance, and on condition of privacy, seeming to fear that there might be sufficient grounds for classifying him with the ruminant order of animals.

He says the process commenced when he was about ten years old, when he was in perfect health, and without any assignable cause. The regurgitation takes place regularly, after every meal, commencing about fifteen minutes after finishing the meal. The returning bolus is remasticated, and again swallowed. It does not, however, return to the mouth in a distinctly-molded form, as in those animals whose organs are adapted to the special and normal function of rumination, but is, rather, a loose, discrete mass.

The act is repeated in the same way, the frequency and the length of time it continues depending on the quantity, the kind, and condition, of food taken.

When a light meal is taken, consisting largely of liquids, there may be but one or two regurgitations following. When a meal consists, to any extent, of meats, and the coarser vegetables, containing considerable of the fibrous elements, as might be inferred, the acts of regurgitation are more frequent, and continue longer, until, sometimes, from three to three and a half hours intervene between the meal and the last act of regurgitation. Although he thinks he chews his food as well, and with as much care, as other people, yet, when he gives particular attention to it, and masticates thoroughly, it notably diminishes the number and frequency of the acts of regurgitation.

When asked how far the process was voluntary, he replied, that he could control it, to some extent, by keeping his mind directed to it; but, when it is repressed, it causes an uncomfortable and peculiar feeling of heaviness, somewhat resembling slight nausea.

There is concerned, in each act, the element of a slight contraction of the muscles of the abdominal cavity, which is, to some extent, voluntary; but almost unconsciously so.

The taste of the regurgitated food is not modified during the first part of the process; but, towards the latter part, it acquires a disagreeable acid taste. But, to use his own words, chewing the regurgitated food, before it has acquired this disagreeable taste, "is the sweetest part of the meal."

It causes him no inconvenience or disturbance of any kind, except some embarrassment, during the process, in presence of others, and the (to him) disagreeable reflection that it is abnormal, and not human.

These are the principal facts in the case, as I learned them from his own statements and from personal observation; and I would respectfully submit them to the Society, soliciting discussion on the questions that must naturally arise as to the difficulty, nature and cause of the difficulty, and means of relief.

